# Congenital Malformation

**Published:** 1878-12-20

**Authors:** L. G. Hardman

**Affiliations:** Ga.


### CONGENITAL MALFORMATION

BY L. G. HARDMAN, OF GA.

As the post mortem examination of this case reveals something that is not very common, it will be perhaps of some interest to your readers, and therefore I offer it for publication. I shall not, however, give in full the particulars of the case.

The case was an infant five days old, who had been operated upon for imperforate anus, but without success.

On the 8th day of July the infant died, and the examination revealed the following conditions: No communication between the jejunum and ilium; deficient rectum and double uterus. The jejunum terminated in a pouch, or cul-de-sac, and was joined to the ilium by connective tisue. The pouch was distended with milk which had not been vomited.

Passing down the bowels, I found nothing wrong until I came to the sigmoid fluxure, which terminated in a pouch a little below the promontory of the sacrum, and firmly adhered to it.

The next thing which attracted attention was a peculiar uterus. After examining, found it to be a double uterus, with only one fallopian tube to each, and passing off from the fundus in a straight line; or, in other words, the fundus of each terminated in a fallopian tube.

To give a more definite idea of this peculiar deformity, I will compare it to the hind legs of the frog, separated from the body at the lumbar region. The legs represent the fallopian tubes, and the toes the fembriated extremities of the same, while the sacrum and lumbar vertebra represent the body and cervix of the uterus as they are attached to each other.

Although malformations of this organ are not very rare, I do not find any thing of this kind on record.

We find in the old works of obstetrics great many malformations, such as double uterus, but quite different from this specimen. It is said that the uterus in this malformation resembles that of some lower animal. Whether this be true or not I am not able to say. This case is, however, peculiar in the number of malformations in the same body—the other organs of the body being normal. I have this specimen in my office, and if any one feels interested, I will give them any information concerning it I can.

